# The Book World of Medicine and Science

**Published:** 1896-04-04

**Authors:** 


### The Book World of Medicine and Science.

Early Scoliosis, or Curable Curvatures of the Spine. By Percy G. Lewis, M.D., M.R.C.S.Eng., &c. (London: John Bale and Sons, 85 and 89, Great Titchfield Street, Oxford Street, W. 49pp. 10 illustrations. 1895.)

The author states in his preface that "this little book has its origin in the difficulty he experienced, on first entering general practice, in diagnosing and treating these cases," and that it is merely intended as a guide to young practitioners who may experience similar difficulties. From this standpoint it is an eminently useful addition to orthopædic literature, for an early diagnosis of spinal curvatures is not only of the first importance for effective treatment but at the same time often beset with considerable difficulty.

In discussing the anatomical changes the action of the muscles giving rise to the rotation is dwelt upon, and the fact, often overlooked, that the line represented by the tips of the spinous processes does not give any actual idea of the extent of the curvature, is explained.

In dealing with etiology, among bad positions, resting the weight of the body on one leg while standing, and writing at too high a table are mentioned, as well as the necessity of counteracting the effects of flat-foot, knock-knee, shortened limbs, &c. The author also speaks of the bad effects of ill-fitting coats and dresses, which are almost invariably made too tight in front. He omits, however, to mention short-sight as a most important factor, and also the stupid system now happily fast disappearing from our schools of insisting on a slanting form of writing whereby the body must necessarily be placed in a twisted position. Schoolmasters and mistresses are still very largely responsible for the numbers of cases of spinal curvatures met with in this country.

The author very rightly emphasises the necessity for early diagnosis and treatment before the ligamentous and osseous changes have made an absolute cure impossible, he describes the methods used by orthopædic surgeons for ascertaining the extent of the rotation, and differentiates between the symptoms of caries and scoliosis.

In the matter of treatment, fresh air, tonics, &c., are dwelt upon, and a large part of the book is devoted to the description of the author's vertical bolster, and spinal chair, of which illustrations are given, as well as photographs showing the different uses of Dowd's exercising machine. These exercises seem very suitable for some cases, but in the earlier stages it is doubtful whether regular harmonious exercises in the recumbent position would not be more suile tab for atonic patients. He very truly condemns ordinary scholastic gymnastics in cases of actual curvature, however useful they may be as a preventive measure, and remarks that "success is greatest to him who knows how to vary his prescriptions and instructions for the general mode of life to each case." His experience accords with that of orthopædic surgeons respecting the harmfulness of so-called spinal supports and braces.

The plates are clear and well executed.

A Pharmacopœia for Diseases of the Skin. Edited by James Startin, Senior Surgeon to the London Skin Hospital. (Bristol: John Wright and Co., 1896. Price 2s. 6d.)

This is but a little book, in fact, a very tiny one indeed, weighing just over two ounces, although strongly bound in cloth boards, and we must congratulate its author in having compressed into so small a space the not too weighty knowledge which he cares to convey to the "students and medical practitioners," for whom it professes to be written. Whether it is Mr. Startin's private receipt book, or the pharmacopœia of the Skin Hospital in Fitzroy Square, is nowhere stated, but in neither case can we think very highly of skin specialism if this is all it can show.

A pharmacopœia issued by a special hospital or surgeon may fairly be expected to show the drugs and methods used in the "special" treatment of disease. Yet after all the exaltation of the specialist, as a being superior and apart, when we come to take a peep into his actual laboratory and see the details of that "special" treatment, as the exponent of which he takes so high a place in public estimation, all we find is this wonderfully small bundle of old-fashioned receipts. Of course the answer to all this is that it is by his skill, and the marvellous power of differentiation of conditions, and by his exact adaption of his remedies to these conditions, that the specialist excels, rather than by anything peculiar in the drugs he uses. It may then be well to point out that so far is this from always being the case, that in this charming little guide to treatment all skin diseases are lumped together as regards their dietetics, one set of "Rules of Diet" being given as apparently applicable to all. Even the "medical practitioner who may be unacquainted with the ordinary nomenclature of skin diseases," one of the strange beings who are expected to find this book useful, knows better than that.

The Vaccination Question. By Arthur Wollaston Hutton. (London: Methuen and Co.)

If the vaccination controversy were a mere question of dialectic, Mr. Wollaston Hutton would be well to the front, for he wields a decidedly skilful pen. Unfortunately for him, mere forensic methods have no place in the decision of matters of science. In science, facts form the only proper bases of discussion; and those inferences are alone legitimate which can be deduced, without unnatural strain, from the facts. Tried by these elementary principles of science, nine-tenths of Mr. Hutton's over-laboured argument is promptly put out of court. There are, in a question of this kind, only two points which responsible writers really care to discuss. The first is this—does efficient vaccination really act as a prophylactic agent against small-pox? The second this—assuming that vaccination is an efficient prophylactic against small-pox, is it politically expedient that the whole population shall be compulsorily vaccinated? The honest reasoner, therefore, turns first to that part of Mr. Hutton's work which deals with the question whether vaccination is, or is not, an efficient prophylactic against small-pox. What help does Mr. Wollaston Hutton give us towards the final determining of this question? Absolutely none. His contribution to the literature of the subject is a mere re-hash of the argument of Crookshank and Creighton; and though no doubt his literary skill is equal to that of either of these competent antivaccinalists, one nevertheless misses the touch of the trained man of science, and to that extent one feels that the antivaccination cause, not good in itself, loses by being handled by a layman instead of by a qualified and experienced medical man. As to the question of compulsion, that is another matter. We cannot help feeling, however, that if Mr. Wollaston Hutton and other antivaccinators would frankly admit that the facts as to the value of vaccination are absolutely against them, the public would be much more disposed to consider with attention their contentions in favour of some modification of the compulsion statutes. The best thing that can be said of Mr. Hutton's "Vaccination Question" is that it leaves the controversy exactly where it was before.

An Encyclopædia of Gardening. By T. W. Sanders, F.R.H.S. (W. H. and L. Collingridge, Aldersgate Street, London, E.C. Price 3s. 6d.)

This handy volume contains the common and scientific name of most of the plants in general cultivation. Each plant is treated from the point of view of the soil most suitable to it, and the position in which it is most likely to thrive. Numerous suggestions as to details of culture are given with each plant. As a gardening encyclopædia it will be found a useful companion.

